# Mechanical stiffness promotes skin fibrosis through Piezo1-mediated arginine and proline metabolism

**DOI:** 10.1038/s41420-023-01656-y

**Published:** 2023-09-26

**Authors:** Jiahao He, Bin Fang, Shengzhou Shan, Qingfeng Li

**Affiliations:** Department of Plastic and Reconstructive Surgery, Shanghai Ninth People’s Hospital, Shanghai Jiao Tong University School of Medicine, 200011 Shanghai, China

**Keywords:** Mechanisms of disease, Metabolic diseases

## Abstract

The increased mechanics of fibrotic skin tissue continuously regulate fibroblast functions such as survival and differentiation. Although all these processes consume metabolites, it is unclear whether and how cells adapt their metabolic activity to increased matrix stiffness. Here, we show that transferring mouse dermal fibroblasts from soft to stiff substrates causes an up-regulation of arginine and proline metabolism. Increased matrix stiffness stimulates the expression and activity of key metabolic enzymes, leading to the synthesis of L-proline, a major source of collagen. In addition, the novel mechanosensitive channel Piezo1 was identified as a key regulator of arginine and proline metabolism in fibroblasts under increased stiffness. Consistently, targeting Piezo1 to dermal fibroblasts in vivo effectively reduces fibrosis and arginine-proline metabolism in mouse skin. Therefore, mechanical stiffness is a critical environmental cue for fibroblast metabolism and skin fibrosis progression.

## Introduction

Excessive extracellular matrix (ECM) deposition is a hallmark of fibrotic skin diseases such as hypertrophic scars, keloids, scleroderma and fibrosis induced by surgery, radiotherapy or drugs [1, 2]. Excessive production, remodeling and cross-linking of ECM contribute to the increased matrix stiffness in skin fibrosis [3]. Importantly, aberrant matrix stiffness induces terminal differentiation of fibroblasts into myofibroblasts, which secrete additional ECM components [4]. However, molecular mechanisms that link ECM stiffness and myofibroblast activation remain elusive.

Numerous studies have pointed out that metabolic activities are necessary for fibroblasts functions [5, 6]. In fibroblasts biology, the perturbation of fatty acid oxidation (FAO) and glycolysis leads to ECM production and degradation [7]. Myofibroblasts upregulate key glycolytic enzymes, such as lactate dehydrogenase and hexokinase 2 (HK2), which promote proliferation and collagen synthesis [8, 9]. Glycolysis perturbation has been implicated in lung [10], liver [11] and kidney fibrosis [12], and inhibition of glycolysis inhibits ECM deposition [13]. Fibroblasts also exploit glucanolytic reprogramming to stabilize collagen [14]. Overall, key metabolic pathways including amino acid and lipid metabolism are identified as important drivers of myofibroblast activation. Thus, reversing metabolic reprogramming has emerged as a promising strategy to reduce myofibroblast activation across various organs [15].

Recently, it is reported that mechanical stiffness from the microenvironment could continuously modulate cell functions via metabolic pathways [16–18]. Epithelial cells sense the mechanical stiffness by actomyosin cytoskeleton to maintain the persistence of high glycolytic rates [18]. Dendritic cells (DCs) cultured under higher mechanical stiffness exhibit increased activation and glycolytic flux [19]. In lung cancer progression, the mechanosensitive kindlin-2-pyrroline-5-carboxylate reductase 1 (PYCR1) complex could link mechanical stiffness to proline metabolism [20]. In stem cells adipogenic differentiation, soft matrix stiffness reinforces lipid synthesis and storage by activating SREBP [21]. Several mechanosensitive factors, such as integrins [22], focal adhesions [23, 24] and yes-associated protein (Yap) [25], are important regulators in myofibroblasts activation. Those mechanosensors are also key factors mediating metabolic activity to mechanical stiffness in other cell types [19, 26]. However, we still lack an understanding of how mechanics-induced metabolic program may modulate myofibroblasts activation. In 2010, the Piezo1 protein was identified as a member of a novel class of mechanically activated ion channels [27]. Piezo1 is expressed in a wide range of tissues and is involved in many physiological functions [28]. The novel mechanosensitive channel Piezo1 has been shown to directly sense matrix stiffness. In neural stem cell differentiation, Piezo1 provides a link between ECM mechanics and intracellular signaling [29]. Monocytes also regulate angiogenesis via Piezo1 based on the mechanical properties of their surrounding matrix [30]. In particular, in dendritic cells, Piezo1 serves as an important sensor that promotes cell metabolism and inflammatory function under environmental stiffness [19]. As mechanical stiffness has emerged as an important driver of cellular metabolism, we postulated that the Piezo1 channel may be a key mediator involved in the induction of fibroblast metabolism by mechanical signals.

Here we show that mechanical stiffness is a critical environmental cue that affects the metabolism and function of dermal fibroblasts. Mouse dermal fibroblasts (MDFs) grown under increased matrix stiffness exhibit myofibroblast differentiation and a greater capacity to promote tissue fibrosis in mice. Metabolomics analysis revealed that MDFs cultured at higher tension showed upregulation of the arginine and proline metabolism pathway, which are sources for collagen biosynthesis. The pathway works by inducing the expression and activation of metabolic enzymes known to increase arginine and proline metabolism. In addition, the mechanosensitive channel Piezo1 was identified as a mechanosensor in MDFs arginine and proline metabolism in vitro and in vivo. Our results demonstrate a critical role for mechanical stiffness in controlling metabolic processes in dermal fibroblasts.

## Result

### Increased matrix stiffness promotes MDFs differentiation

**Fig. 1:**
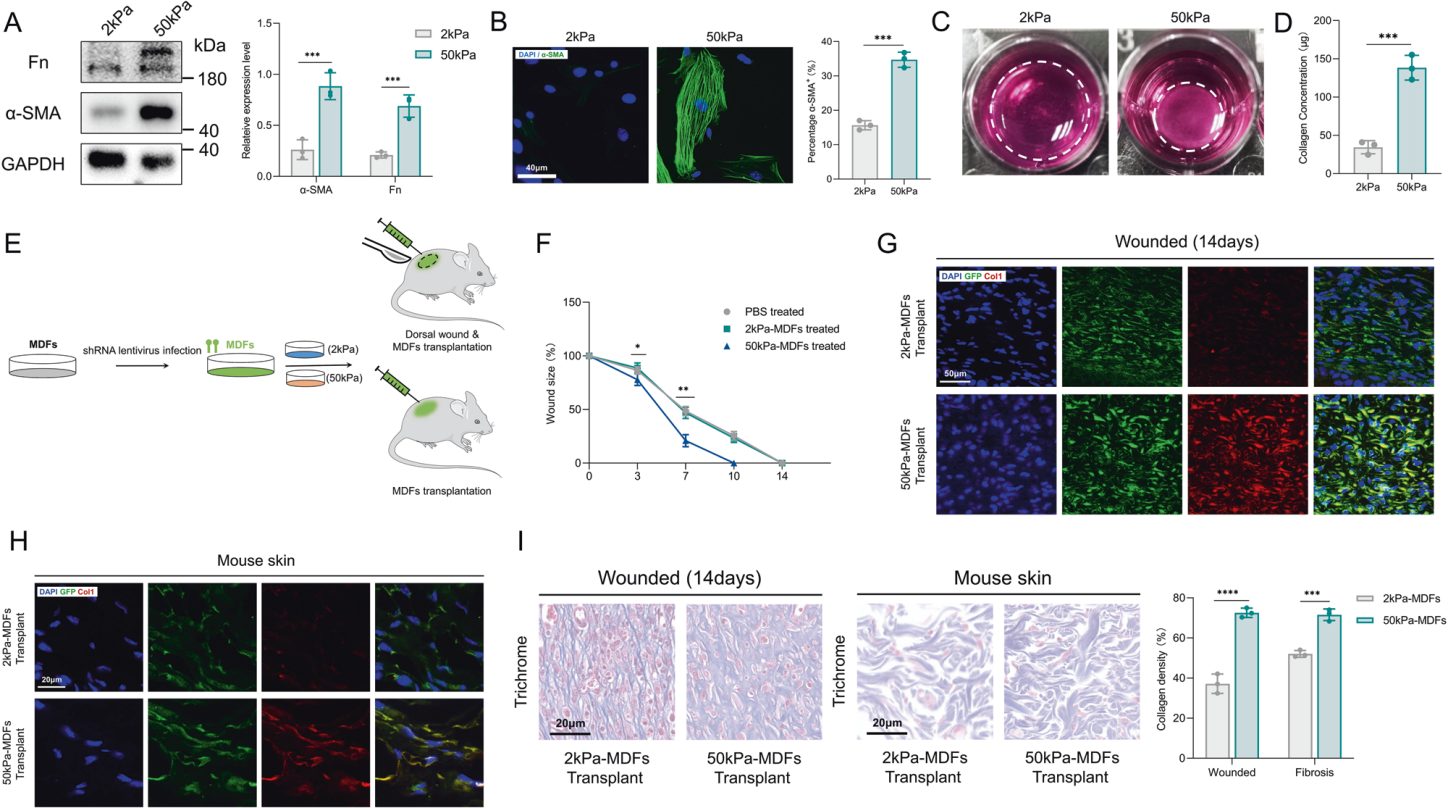
MDFs Function in different stiffness substrate in vitro and in vivo. **A** Western blot assay for α-SMA and Fn in MDFs on 2 versus 50 kPa hydrogel and quantitative analysis (*n* = 3). **B** Soluble collagen concentration of MDFs on 2 versus 50 kPa hydrogel (*n* = 3). **C** Immunofluorescence staining for α-SMA of MDFs on 2 versus 50 kPa hydrogel and quantitative analysis (*n* = 3). **D** Collagen contraction assay of MDFs on 2 versus 50 kPa hydrogel (*n* = 3). **E** Schematic depicting MDFs labeling, culture and transplantation in dorsal wounding and ECM deposition experiments. **F** Quantitative analysis of wound area. Percentage means day 0 wound size versus days since wounding (*n* = 10). **G** Imaging of transplanted GFP-positive 2 kPa or 50 kPa MDFs in wounding and colocalization between GFP and or Col-I signaling. **H** Imaging of transplanted GFP-positive 2 kPa or 50 kPa MDFs in skin fibrosis and colocalization between GFP and or Col-I signaling. **I** Trichrome staining of 2 kPa and 50 kPa MDFs transplanted wounds or mouse skin (*n* = 3). The results are expressed as the means with SD. ***P < 0.005, ****P < 0.001.

To investigate the effects of increased matrix stiffness on the phenotypes of MDFs, we cultured cells on polyacrylamide hydrogel plates of 2 kPa and 50 kPa Young’s modulus, mimicking the stiffness observed in normal and fibrotic skin, respectively. Exposure of MDFs to increased stiffness induced their differentiation into myofibroblasts, characterised by α-smooth muscle actin (α-SMA) expression (Fig. 1A, B). The collagen gel contraction assay confirmed that increased stiffness enhanced contraction of MDFs through α-SMA synthesis (Fig. 1C). Cells grown on increased stiffness also exhibited higher collagen and fibronectin (Fn) secretion (Fig. 1A, D). Furthermore, we used MDFs transplantation assays to investigate collagen secretion of 2 kPa/50 kPa cultured MDFs in vivo. GFP lentivirus-tagged MDFs were cultured on 2 kPa and 50 kPa substrates and then transplanted intradermally into the dorsal wound or dermis of mice (Fig. 1E). Wounds transplanted with 50 kPa MDFs showed faster wound healing rates (Fig. 1F). Consistent with our in vitro studies, stiff substrate increased collagen production of MDFs in vivo, by simultaneous GFP/Col1 staining of skin sections and trichrome staining (Fig. 1G–I). Thus, these results suggest that increased matrix stiffness drives MDFs differentiation into myofibroblasts, particularly by upregulating ECM production.

### Increased matrix stiffness promotes MDFs arginine and proline metabolism in vitro

**Fig. 2:**
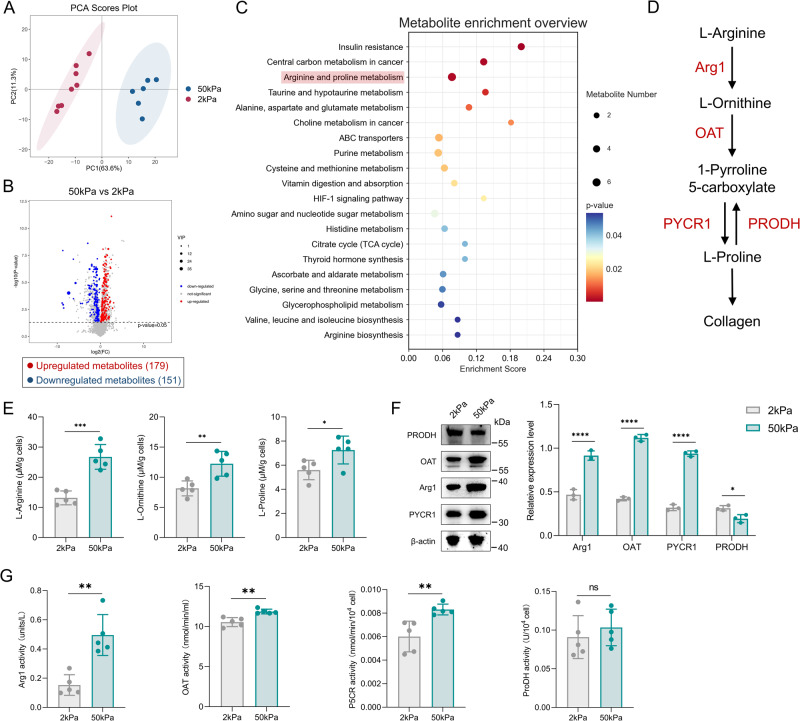
Metabolomics confirm increased matrix stiffness drive MDFs arginine and proline metabolism. **A** Principal component analysis showed separation of 50 kPa-MDFs (*n* = 6) vs 2 kPa-MDFs (*n* = 8). **B** Volcano map between 50 kPa-MDFs and 2 kPa-MDFs. **C** Metabolites enrichments for significantly upregulated pathways from 50 kPa-MDFs vs 2 kPa-MDFs. **D** Illustration schematic of arginine and proline metabolism pathway. **E** Measurement of L-Arginine, L-Ornithine and L-Proline in 2 kPa-MDFs and 50 kPa-MDFs (*n* = 5). **F** Western blot assay for key metabolic enzymes Arg1, OAT, PYCR1 and PRODH in 2 kPa-MDFs/50 kPa-MDFs and quantitative analysis (*n* = 3). **G** Measurement of key metabolic enzymes activity Arg1, OAT, PYCR1 and PRODH in 2 kPa-MDFs/50 kPa-MDFs and quantitative analysis (*n* = 5). The results are expressed as the means with SD. *P < 0.05, **P < 0.01, ***P < 0.005, ****P < 0.001.

Fibroblast activation are accompanied by sustained metabolic reprogramming [31]. To obtain an overview of the metabolic changes in MDFs at different matrix stiffnesses, we screened MDFs cultured on 50 versus 2 kPa substrates using metabolomic analysis. Using principal component analysis, MDFs grown on 50 kPa clustered separately from those grown on 2 kPa (Fig. 2A). Compared to MDFs cultured on soft substrates, MDFs cultured on stiff substrates showed an enrichment for “arginine and proline metabolism” (Fig. 2B–D). The accumulation of L-proline, a necessary precursor for collagen synthesis, strongly suggested that stiff substrates enhanced ECM production through L-proline anabolism in MDFs. LC-MS/MS confirmed the systematic up-regulation of L-arginine, L-ornithine and L-proline when cells were cultured on stiff substrates (Fig. 2E). To gain insight into the effect of stiff substrates on L-proline anabolism, we next looked for changes in relevant metabolic enzymes. The expression of Arg1, OAT and PYCR1, which control L-proline anabolism, is upregulated in stiff substrate (Fig. 2F). Consistent with the increased expression of these metabolic enzymes, MDFs on stiff substrate showed higher metabolic enzyme activity (Fig. 2G). In addition, the expression of the L-proline catabolic enzyme PRODH was downregulated in the stiff substrate (Fig. 2G). The activity of PRODH remained unchanged on stiff substrate. Collectively, these data suggest that MDFs possess the machinery to transduce extracellular mechanical signals into intracellular proline metabolism to promote its activation.

### Increased matrix stiffness promotes MDFs arginine and proline metabolism in vivo

**Fig. 3:**
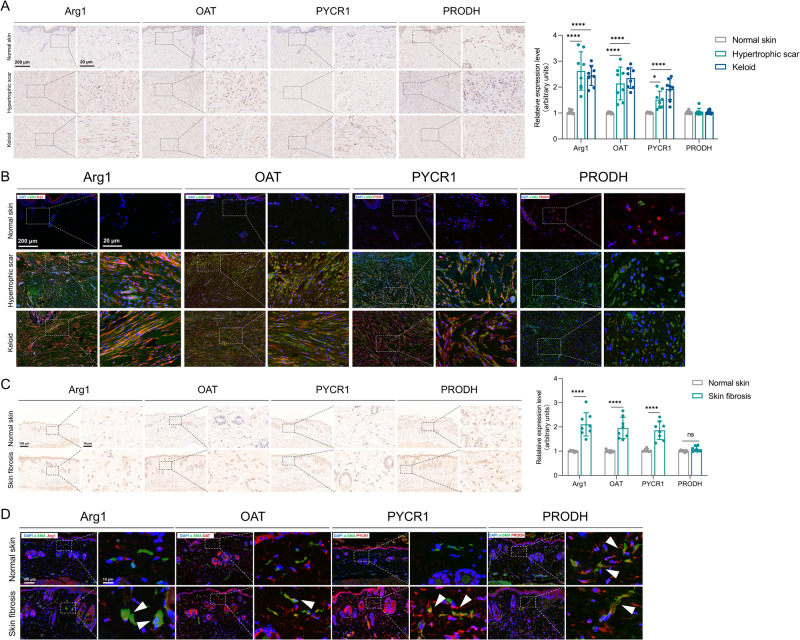
Detection of key metabolic enzymes in human and mouse samples. **A** Immunostaining of Arg1, OAT, PYCR1 and PRODH in human normal skin, hypertrophic scar and keloid tissues and quantitative analysis (*n* = 8). **B** Colocalization of Arg1, OAT, PYCR1, PRODH with α-SMA in human normal skin, hypertrophic scar and keloid tissues. **C** Immunostaining of Arg1, OAT, PYCR1 and PRODH in mouse normal skin and skin fibrosis tissues (*n* = 8). **D** Colocalization of Arg1, OAT, PYCR1, PRODH with α-SMA in mouse normal skin and skin fibrosis tissues. The results are expressed as the means with SD. ****P < 0.001.

Changes in dermis mechanics occur during skin fibrosis [32]. We therefore hypothesized that abnormal matrix stiffness in fibrotic dermis affects arginine and proline metabolism in fibroblasts in vivo. We observed that human fibrotic tissues (hypertrophic scar and keloid) have higher expression of Arg1, OAT and PYCR1 than normal skin (Fig. 3A). The expression of PRODH was unchanged. Immunofluorescence staining confirmed the up-regulation of Arg1, OAT and PYCR1 in dermal myofibroblasts (Fig. 3B). Fibrosis has been reported to increase skin stiffness in a mouse model of scleroderma. Our data show that Arg1, OAT and PYCR1 expression were increased in the bleomycin dermal fibrosis model (Fig. 3C). Consistent with human fibrotic tissues, mouse dermal myofibroblasts also show higher Arg1, OAT and PYCR1 expression (Fig. 3D). Thus, these results suggest that mechanical changes to the dermis drive arginine and proline metabolism in myofibroblasts in vivo.

### Mechanosensors Piezo1 impacts MDFs arginine and proline metabolism in vitro

**Fig. 4:**
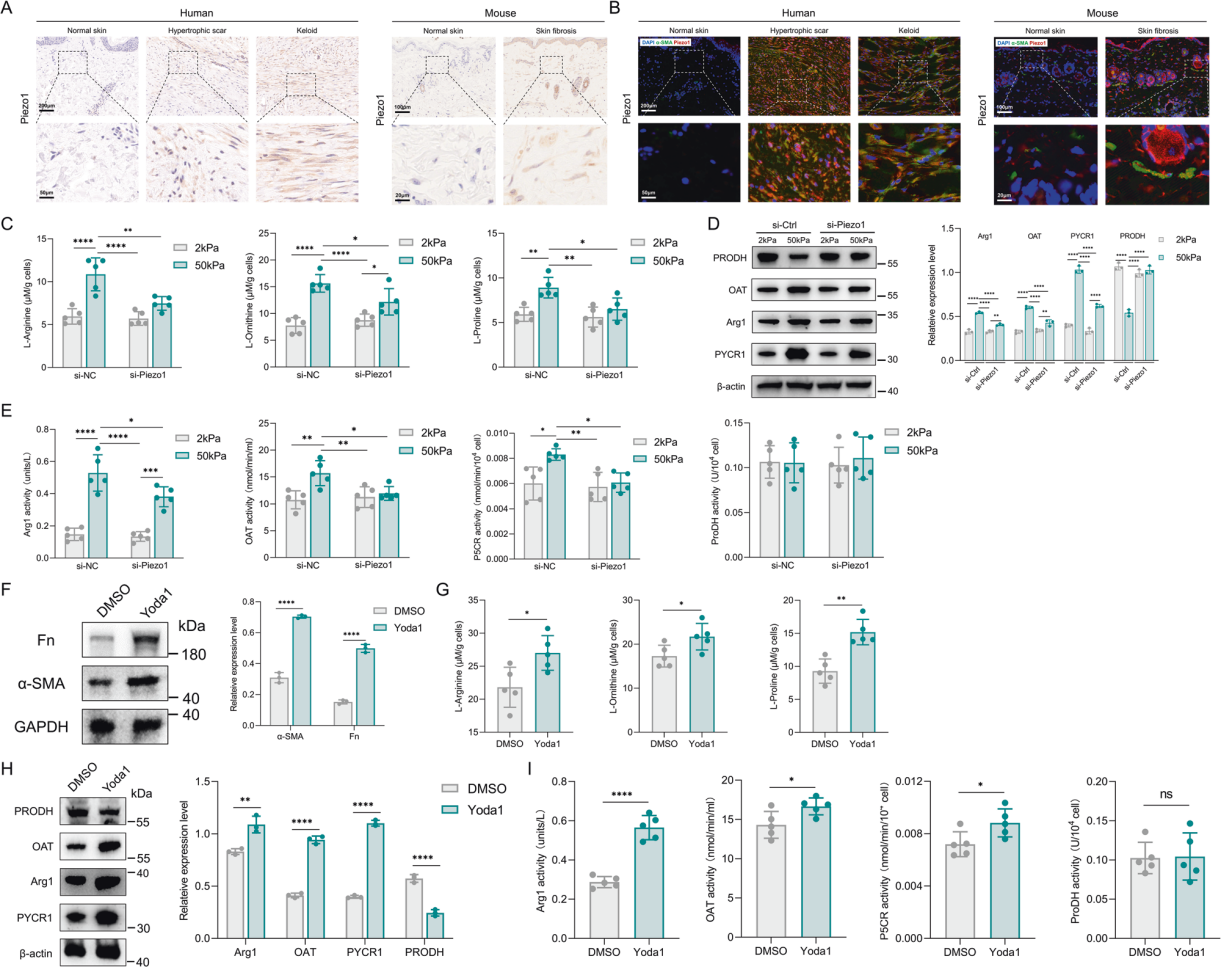
The role of Piezo1 in increased matrix stiffness-induced MDFs arginine and proline metabolism in vitro. **A** Piezo1 expression in human normal skin, hypertrophic scar, keloid (Scale bar = 200 μm, Zoom scale bar = 50 μm) and mouse normal/fibrotic skin (Scale bar = 100 μm, Zoom scale bar = 20 μm). **B** Images of immunofluorescence co-staining of Piezo1 (red) and α-SMA (green) in human normal skin, hypertrophic scar, keloid (Scale bar = 200 μm, Zoom scale bar = 50 μm) and mouse normal/fibrotic skin (Scale bar = 100 μm, Zoom scale bar = 20 μm). **C** Measurement of L-Arginine, L-Ornithine and L-Proline in 2 kPa-MDFs/50 kPa-MDFs with or without Piezo1 knockdown (*n* = 5). **D** Western blot assay for key metabolic enzymes Arg1, OAT, PYCR1 and PRODH in 2 kPa-MDFs/50 kPa-MDFs with or without Piezo1 knockdown and quantitative analysis (*n* = 3). **E** Measurement of key metabolic enzymes activity Arg1, OAT, PYCR1 and PRODH in 2 kPa-MDFs/50 kPa-MDFs with or without Piezo1 knockdown and quantitative analysis (*n* = 5). **F** Western blot assay for α-SMA and Fn in MDFs with or without Yoda1 (25 μM) and quantitative analysis (*n* = 3). **G** Measurement of L-Arginine, L-Ornithine and L-Proline in MDFs with or without Yoda1 (25 μM) (*n* = 5). **H** Western blot assay for key metabolic enzymes Arg1, OAT, PYCR1 and PRODH in MDFs with or without Yoda1 (25 μM) and quantitative analysis (*n* = 3). **I** Measurement of key metabolic enzymes activity Arg1, OAT, PYCR1 and PRODH in MDFs with or without Yoda1 (25 μM) and quantitative analysis (*n* = 5). The results are expressed as the means with SD. *P < 0.05, **P < 0.01, ****P < 0.001.

Piezo1, a Ca^2+^-dependent mechanosensitive channel, was recently shown to mediate bone anabolism [33] and iron metabolism [34], and it plays a crucial role in fibroblasts mechanical signal transduction. Firstly, we investigated the protein level of Piezo1 in human fibrotic tissues (hypertrophic scar and keloid) and mouse model of bleomycin-induced skin fibrosis. Immunohistochemical analysis confirmed an increase in Piezo1 in human and mouse fibrotic skin tissues (Fig. 4A). As shown by α-SMA/Piezo1 immunofluorescence co-staining, we observed that Piezo1 is highly expressed in myofibroblasts in human and mouse fibrotic skin tissues (Fig. 4B). To evaluate a role for Piezo1 in the arginine and proline metabolism of MDFs in vitro, we tested the effect of a Piezo1 siRNA on MDFs cultured on 50 kPa substrates. The levels of L-arginine, L-ornithine and L-proline were downregulated in Piezo1 knockdown MDFs grown on 50 kPa substrates (Fig. 4C). Piezo1 knockdown inhibited the increased Arg1, OAT and PYCR1 expression and rescued PRODH expression when the cells were grown on stiff substrate (Fig. 4D). Piezo1 knockdown also effectively reduces stiff substrate-mediated Arg1, OAT and PYCR1 activity (Fig. 4E). To assess a role for Piezo1 in MDFs metabolism in vitro, we tested the effect of a Piezo1 agonist, Yoda1 on MDFs. Yoda1 stimulated MDFs activation and upregulated the production of L-arginine, L-ornithine and L-proline (Fig. 4F, G). The addition of Yoda1 also induced the expression of Arg1, OAT and PYCR1 (Fig. 4H). We also observed that Yoda1 stimulated Arg1, OAT and PYCR1 activity (Fig. 4I). Taken together, Piezo1 could potentially contribute to the mechanosensory pathways that support arginine and proline metabolism in MDFs in vitro.

### Mechanosensors Piezo1 impacts MDFs arginine and proline metabolism in vivo

**Fig. 5:**
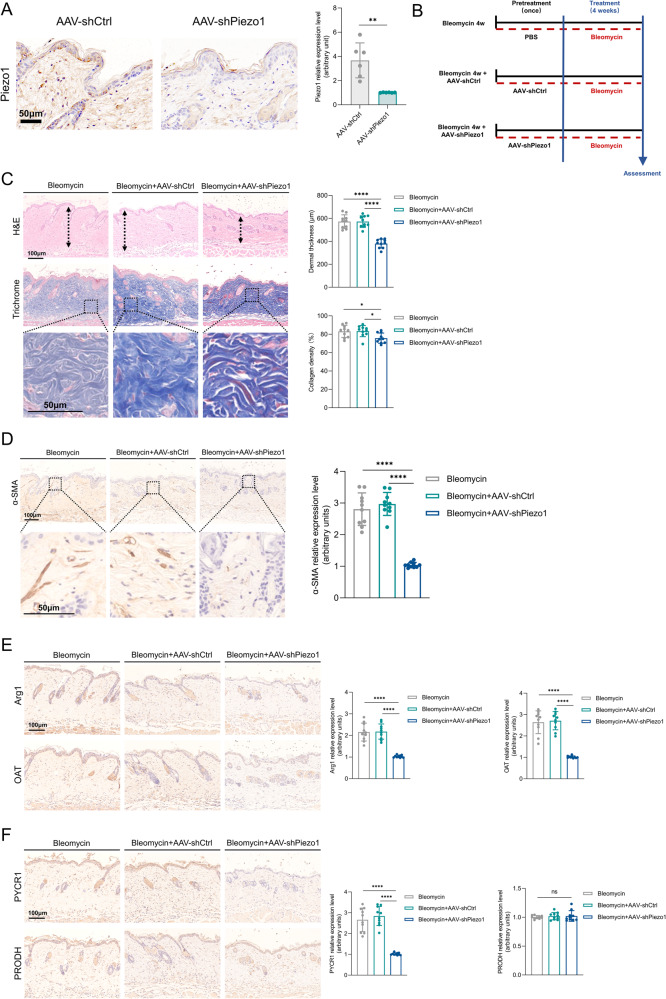
The role of Piezo1 in increased matrix stiffness-induced MDFs arginine and proline metabolism in vivo. **A** Validation of Piezo1 knockdown in mouse skin and quantitative analysis of Piezo1 staining (*n* = 6). **B** Schematic illustration of the experimental procedure. **C** Representative images and quantitative analysis of H&E and Trichrome staining (*n* = 10). **D** Representative images and quantitative analysis of α-SMA staining (*n* = 10). **E** Representative images and quantitative analysis of Arg1 and OAT staining (*n* = 10). **F** Representative images and quantitative analysis of PYCR1 and PRODH staining (*n* = 10). The results are expressed as the means with SD. *P < 0.05, **P < 0.01, ****P < 0.001.

To investigate the effect of Piezo1 on MDFs metabolism in vivo, we used AAV9-shPiezo1 to knock down Piezo1 expression in mouse skin (Fig. 5A). We then established the bleomycin-induced skin fibrosis model (Fig. 5B). AAV9-shPiezo1 significantly reduced bleomycin-induced dermal fibrosis compared to AAV9-shCtrl treatment, as assessed after 4 weeks by H&E and trichrome staining of skin, measurement of skin thickness and collagen density (Fig. 5C). As shown by α-SMA staining, AAV9-shPiezo1 treatment also reduced the number of myofibroblasts in the bleomycin-treated mouse (Fig. 5D). Consistent with our in vitro studies, Piezo1 knockdown decreased the expression of Arg1, OAT and PYCR1 in the dermis region (Fig. 5E, F. Taken together, these results suggest that Piezo1 promotes matrix stiffness-mediated arginine and proline metabolism of MDFs in vivo.

## Discussion

Here we demonstrate a potential link between mechanical signaling, metabolism and cell function in dermal fibroblasts. By comparing MDFs grown on stiff (50 kPa) versus soft (2 kPa) hydrogel substrates, we reported that increased substrate stiffness conferred an enhanced fibroproliferative phenotype. MDFs responded to increased substrate stiffness by metabolic reprogramming. Higher stiffness upregulated arginine and proline metabolism in MDFs to stimulate their activation as reflected by increased metabolites, metabolic enzyme gene expression and metabolic enzyme activity. Interestingly, we also observed that the mechanosensitive channel Piezo1 mediated metabolic reprogramming of MDFs and Piezo1 knockdown mice lost a skin fibrosis phenotype with reduced arginine and proline metabolism.

Changes in dermal mechanics occur during skin aging and fibrosis and influence the activation of resident fibroblasts [32]. Consistently, fibroblasts respond to the stiffness of their substrate and determine cell differentiation and gene expression [35]. Although these processes require metabolites, it is unknown whether and how fibroblasts adapt their metabolic activity to sense substrate stiffness. We report that mechanical stiffness controls the activation of MDFs by stimulating arginine and proline metabolism. L-arginine and its metabolite L-ornithine are catabolized by the key metabolic enzyme, which is further metabolized to L-proline, which is essential for the synthesis of collagen [36]. Although this metabolic process is well recognized in fibrosis [37] and tumorigenesis [38, 39], it remains to be determined in stiffness-mediated fibroblast activation. Previous studies have shown that cyclic stretch appears to promote collagen synthesis by regulating L-arginine metabolism [37]. Increased matrix stiffness is intrinsically linked to up-regulation of proline synthesis via the kindlin-2 and PYCR1 complex, which in turn promotes collagen synthesis, cell survival and tumor progression [20]. Thus, our findings raise the question of whether stiffness-mediated arginine and proline metabolism in fibroblasts leads to tissue fibrosis. There is increased expression of key metabolic enzymes (Arg1, OAT and PYCR1) in fibrotic tissue and cancer. Our data showed that key metabolic enzymes (Arg1, OAT and PYCR1) are upregulated in fibroblasts from fibrotic tissues such as human and mouse skin fibrosis samples, suggesting that aberrant stiffness promotes arginine and proline metabolism in vivo. Taken together, our research has shown that matrix stiffness may promote arginine and proline metabolism in fibroblasts, leading to tissue fibrosis.

Next, we identified the mediator by which fibroblasts use mechanical stiffness to regulate their metabolic process. In particular, the novel mechanosensitive channel Piezo1 emerged as a potential mechanistic factor [40]. Piezo1 is important in transmitting increased matrix stiffness to resident cells to modulate cell behavior, for example in Alzheimer’s disease [41], glioma [42] and the ageing brain [43]. However, it is not known whether Piezo1 is involved in the stiffness-mediated metabolic process. Piezo1 has been reported to link matrix stiffness to dendritic cell metabolism and function [19]. In our research, Piezo1 mediated matrix stiffness-induced arginine and proline metabolism in fibroblasts. Since matrix stiffness serves as a regulator of cell metabolism, we believe that Piezo1 may transduce mechanical stimuli in these processes. We and others have shown that Piezo1 can mediate tissue fibrosis induced by mechanical forces, including stretch [24], pressure [44] and matrix stiffness [45]. Now, we demonstrated that Piezo1 might interact with mechanical stiffness to regulate skin fibrosis through metabolic reprogramming of fibroblasts, providing new insights into mechanical force-induced skin fibrosis. However, there might be a complex mechanism underlying Piezo1-mediated fibroblasts metabolism. It will be of great interest to further discover how Piezo1 activity contributes to arginine and proline metabolism in fibroblasts. Overall, our research not only indicate the role of Piezo1 in skin fibrosis, but also reveals that Piezo1 may serve as an important regulator of mechanical force-induced cell metabolic reprogramming.

Taken together, these findings provide a mechanism for understanding how mechanical stiffness triggers metabolic reprogramming to induce skin fibrosis. More broadly, these results suggest that targeted strategies to uncouple mechanical stiffness from the metabolic process and Piezo1 may prove successful in treating fibrosis in various tissues.

## Methods

### Patient samples

Detailed patient information is summarized in Supplementary Table 1. All samples were obtained from Shanghai Ninth People’s Hospital with ethical approval from the local human research ethics committee of Shanghai Jiao Tong University School of Medicine in accordance with the tenets of the Declaration of Helsinki.

### Isolation and culture of mouse dermal fibroblasts (MDFs)

The epidermis was removed from the dorsal skin of newborn mice overnight at 4 °C with 0.2% Dispase II solution (Sigma-Aldrich, St Louis, MO, USA). The next day, the dermis was digested with 0.25% collagenase IV solution (Sigma-Aldrich) at 37 °C for 1 h. After filtration, centrifugation and resuspension, MDFs were cultured in Dulbecco’s modified Eagle’s medium (DMEM) (Gibco Life Technologies, Grand Island, NY, USA) supplemented with 10% fetal bovine serum (FBS) (Gibco Life Technologies) and 1% penicillin/streptomycin (Gibco Life Technologies) at 37 °C in 5% CO2. Passage 3-5 MDFs were used for subsequent experiments.

### Polyacrylamide hydrogel-coated plates

To test the effect of matrix stiffness on MDFs behaviors, cells were seeded on collagen-coated polyacrylamide hydrogels with different stiffnesses (2 kPa and 50 kPa) for 3 days. The Young modulus of plates was in line with previous report [19].

### Green fluorescent protein (GFP) lentivirus labeling MDFs

The GFP reporter vector lentivirus (GenePharma Inc, Shanghai, China) was purchased and dissolved in medium. Briefly, 5 × 10^5^ cells in 6-well plates were transduced with the lentiviral vectors (multiplicity of infection [MOI] of 20) and 5 μg/mL polybrene (GenePharma Inc) at 37 °C overnight. The medium was then aspirated and replaced with fresh medium. After 72 h, MDFs were collected for animal experiments.

### Adeno-associated virus (AAV) vector construction

The construction of the vector carrying the shRNA (against Piezo1 and no-targeting scramble) and its packaging into AAV-9 were carried out by Vector Builder (vectorbuilder.com) at final titer of AAV9 to >1.0 × 10^12^ GC/ml as previously described [46]. The interference sequences were as follows, AAV9-shCtrl: 5′-CCTAAGGTTAAGTCGCCCTCG-3′, AAV9-shPiezo1: 5′-CTGCTATCAGACACCATTTAT-3′. 100 μL (1.0 × 10^11^ GC/ml) of AAV9-shPiezo1 or AAV9-shCtrl was injected into the subcutaneous of mice dorsal skin. After 21 days, mice dorsal skin was collected and performed immunostaining analysis of Piezo1 to confirm whether the target gene has been knocked down.

### siRNA and Transfection

For Piezo1 silencing, MDFs were transfected in 6-well plates with 100 nM Piezo1 siRNA by using Lipofectamine RNAiMAX reagent (Invitrogen, Carlsbad, CA, USA) according to the manufacturer’s protocol. The sequences were as follows: Piezo1-siRNA, 5’-CCGGCAUCUACGUCAAAUATT-3’ (sense) and 5’-UAUUUGACGUAGAUGCCGGTT-3’ (antisense), Control-siRNA, 5’-UUCUCCGAACGUGUCACGUTT-3’ (sense) and 5’-ACGUGACACGUUCGGAGAATT-3’ (antisense). The sequences used were self-selected.

### Animal models of fibrosis

Briefly, 8-week-old C57/BL6 mice (SLAC Laboratory Animal, Shanghai, China) were anaesthetised. 100 μL of either AAV9-shPiezo1 or AAV9-shCtrl was injected subcutaneously into the dorsal skin of the mice 21 days before bleomycin injection. In the bleomycin-induced skin fibrosis model, skin fibrosis was induced by subcutaneous injection of bleomycin (Yeasen Biotechnology, Shanghai, China) for 4 weeks. All animal procedures were performed in accordance with the guidelines of the Animal Care and Use Committee of the School of Medicine, Shanghai Jiao Tong University.

### Collagen gel contraction assay

MDFs were resuspended in collagen gel (Shengyou, Hangzhou, China). Suspension was plated in each well of a 24-well plate. The plates were incubated at 37 °C for 15 min to allow collagen gel polymerization. After gel polymerization, 1 ml DMEM supplemented with 10% FBS was added to each well. The gels were photographed post 48 h.

### Metabolomics

For metabolomics, *N* = 8 biological replicates were sequenced in MDFs grown at 2 kPa and *N* = 6 biological replicates were sequenced in MDFs grown at 50 kPa. The culture medium was completely removed and the cells were immediately placed on liquid nitrogen. Cells were scraped and lysed with ice-cold 80% methanol in water (pre-cooled in a −80 °C freezer). All samples were transferred to tubes and centrifuged at 20,000 × g for 10 min at 4 °C. The supernatant was collected and dried. Metabolites were rehydrated in 100 µl of 0.03% formic acid in liquid chromatography-mass spectrometry (LC-MS)-grade water, vortexed to remove debris, and centrifuged. The supernatant was transferred to a high-performance liquid chromatography (HPLC) vial and metabolite profiling was performed by liquid chromatography-tandem mass spectrometry (LC-MS/MS). LC-MS/MS was performed using a liquid mass spectrometry system consisting of a Dionex U3000 UHPLC high-resolution mass spectrometer and QE plus (Thermo Fisher Scientific). Progenesis Qi V2.3 software (Nonlinear Dynamics, Newcastle, UK) was used to process raw metabolic data after acquisition by Unifi 1.8.1. Differential metabolites were selected according to VIP values and *p*-values obtained from two-tailed Student’s *t*-test of normalised peak areas. Metabolites with VIP values > 1.0 and *p* < 0.05 were considered to be differential metabolites.

### Western blotting

Cells were lysed with ice-cold RIPA buffer and centrifuged at 13,000 g for 15 min at 4 °C. 20 ug of protein lysate (concentration determined by bicinchoninic acid assay) was run on a 10% SDS-PAGE gel at 120 V for 1.5 h and then transferred to nitrocellulose filter membranes (Millipore, Bedford, MA). After blocking with 5% BSA at room temperature (RT) for 1 h, the membranes were incubated with the following primary antibodies: α-SMA (#19245, 1:1000, Cell Signaling Technology, Danvers, MA), fibronectin (ab2413, 1:1000, Abcam, Cambridge, UK), arginase-1 (Arg1) (16001-1-AP, 1: 5000, Proteintech, Wuhan, China), ornithine δ-aminotransferase (OAT) (A6235, 1:1000, ABclonal Technology, Wuhan, China), PYCR1 (13108-1-AP, 1:4000, Proteintech), proline dehydrogenase (oxidase) 1 (PRODH) (22980-1-AP, 1:2000, Proteintech) and β-actin (20536-1-AP, 1:5000, Proteintech) at 4 °C overnight. The next day, the membranes were incubated with HRP-conjugated goat anti-rabbit IgG(H + L) (SA00001-2, 1:10000, Proteintech) for 1 h at room temperature and then washed three times for 10 min with tris-buffered saline containing 0.1% Tween 20. Quantitative analysis was performed on the immunoreactive bands using ImageJ software.

### Soluble collagen detection

Soluble collagen detection was performed using a Sircol® Kit (Biocolor Ltd., Carrickfergus, UK) to quantify the collagen content.

### Yoda1 treatment

MDFs were cultured with or without Yoda1 (Sigma-Aldrich, 25 μM) for 3 days.

### Enzyme activity assay

All enzyme activity assays were performed using enzyme activity kits according to the manufacturer’s instructions. Enzyme activity kits were listed below: Arg1 (MAK112, Sigma-Aldrich), OAT (OAT-2-Y, COMIN, Suzhou, China), PRODH (ProDH-2-Y, COMIN) and P5CR (P5CR-2-W, COMIN). All biological samples were split into five technical replicates.

### Dorsal wounding and transplantation of MDFs

8-week-old C57/BL6 mice were used for cutaneous wound healing experiments. 6-mm full-thickness circular wounds were placed on the middle dorsum of each animal. 2.0 × 10^5^ cells were transplanted via intradermal injection (in 100 μL PBS) into the dorsal wound of mice. Wound areas were harvested after 14 days and processed for analyzation as previously described [47].

### Intradermal transplantation of MDFs

2.0 × 10^5^ cells were transplanted via intradermal injection (in 100 μL PBS) into the dorsal backs of mice. Grafts were harvested after 10 days and processed for analyzation as previously described [47].

### LC-MS/MS for metabolites

Cells were homogenised in chilled 80% (v/v) methanol. Cell lysate samples were centrifuged at 12,000 rpm for 15 min and then transferred to a high recovery glass sample vial for vacuum drying at room temperature. The residue was oximated with 30 μl of pyridine containing 20 mg/ml methoxyamine hydrochloride (Sigma-Aldrich, 226904) at 37 °C overnight and further derivatised with 20 μl of N-tert-butyl dimethylsilyl-N-methyltrifluoroacetamide (Sigma-Aldrich, 394882) at 70 °C for 30 min. The derivatised sample was injected into an Agilent 1290 Infinity LC pump and a 6495 triple quadruple mass spectrometer (Agilent). Standard curves of commercial L-arginine (Sigma-Aldrich, A5006), L-ornithine (Sigma-Aldrich, O2375) or L-proline (Sigma-Aldrich, P0380) were used for quantification in the samples.

### Histology

Samples were fixed with 4% paraformaldehyde overnight, then embedded in paraffin. 5 mm sections were processed and stained with hematoxylin and eosin (H&E) and Masson’s Trichrome Stain Kit following protocol (Solarbio, Beijing, China).

For immunohistochemistry and immunofluorescence, 5 mm sections were incubated with primary antibodies against Piezo1 (ab128245, 1:1000, Abcam), α-SMA (ab7817, 1:200, Abcam), Collagen I (ab6308, 1:200, Abcam), GFP (ab290, 1:500, Abcam), Arg1 (16001-1-AP, 1:400, Proteintech), OAT (A6235, 1:100, ABclonal Technology), PYCR1 (13108-1-AP, 1:200, Proteintech), PRODH (22980-1-AP, 1:400, Proteintech) overnight at 4 °C. Sections were then incubated with Alexa Fluor 594 goat anti-rabbit secondary antibody (125369, 1:200, Jackson lab), and Alexa Fluor 488 goat anti-mouse secondary antibody (133384, 1:200, Jackson lab) for immunofluorescence or an HRP-conjugated goat anti-rabbit secondary antibody (138729, 1:500, Jackson lab) for immunohistochemistry. Images were captured using a Nikon Eclipse E800 microscope (Nikon, Melville, NY, USA).

### Statistical analysis

A two-tailed Student’s *t*-test was used for comparisons between two groups. One-way ANOVA was used for comparing multiple groups. *P* < 0.05 was considered to indicate a significant difference. At least three independent replicates were used for each experiment. Results are expressed as the means ± SD.

### Supplementary information

SUPPLEMENTAL MATERIAL

Original Data File

## Data Availability

All data are available in the main text or the supplementary materials.
