# Supplementary material for: Mechanical stiffness promotes skin fibrosis through Piezo1-mediated arginine and proline metabolism
Source: Cell Death Discov. 2023 Sep 26;9:354. doi: 10.1038/s41420-023-01656-y (PMC10522626; doi:10.1038/s41420-023-01656-y)
Supplement: Supplementary file 1 — SUPPLEMENTAL MATERIAL [file 41420_2023_1656_MOESM1_ESM.docx]

**Supporting Information**

**1. Supplementary Table 1. Patient information**

| Order | Sex | Age (years) | Location |
| --- | --- | --- | --- |
| Hypertrophic scar-1 | Male | 5 | Face |
| Hypertrophic scar-2 | Male | 40 | Neck |
| Hypertrophic scar-3 | Female | 25 | Face |
| Hypertrophic scar-4 | Female | 36 | Neck |
| Hypertrophic scar-5 | Male | 24 | Hand |
| Hypertrophic scar-6 | Female | 20 | Face |
| Hypertrophic scar-7 | Male | 54 | Hand |
| Hypertrophic scar-8 | Female | 32 | Face |
| Keloid-1 | Male | 15 | Chest |
| Keloid-2 | Female | 35 | Chest |
| Keloid-3 | Female | 36 | Abdomen |
| Keloid-4 | Male | 42 | Chest |
| Keloid-5 | Male | 32 | Arm |
| Keloid-6 | Female | 35 | Chest |
| Keloid-7 | Male | 27 | Abdomen |
| Keloid-8 | Female | 30 | Chest |
