# Supplementary figures and images for: Mechanical stiffness promotes skin fibrosis through Piezo1-mediated arginine and proline metabolism
Source: Cell Death Discov. 2023 Sep 26;9:354. doi: 10.1038/s41420-023-01656-y (PMC10522626; doi:10.1038/s41420-023-01656-y)

**Full western blot images**


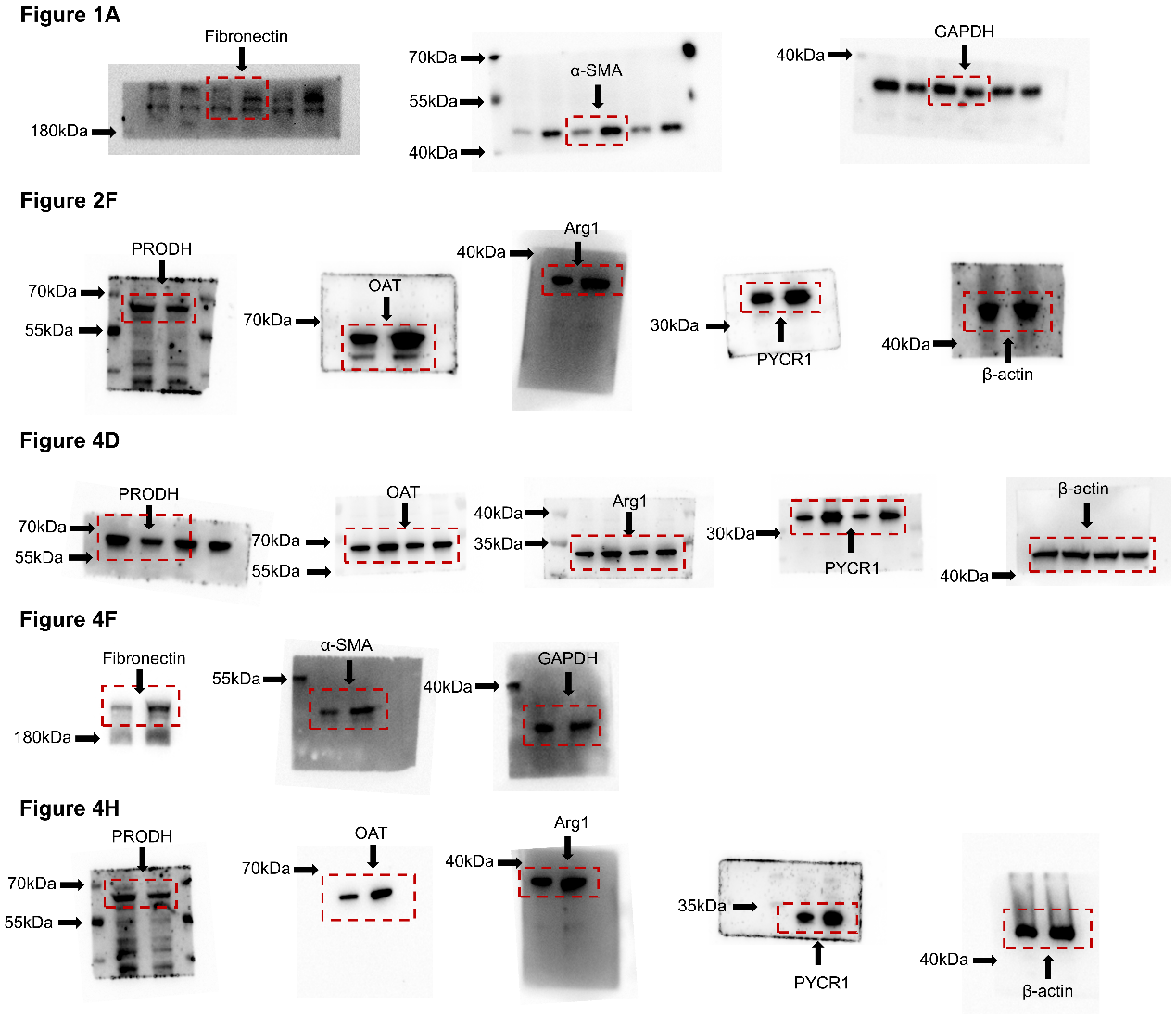

Supplement: Supplementary file 2 — Original Data File [file 41420_2023_1656_MOESM2_ESM.docx]
